# Circulating Vitamin K_1_ Levels in Relation to Ischemic Stroke and Its Subtypes: A Mendelian Randomization Study

**DOI:** 10.3390/nu10111575

**Published:** 2018-10-25

**Authors:** Susanna C. Larsson, Matthew Traylor, Hugh S. Markus

**Affiliations:** 1 Unit of Nutritional Epidemiology, Institute of Environmental Medicine, Karolinska Institutet, SE-171 77 Stockholm, Sweden; 2 Stroke Research Group, Department of Clinical Neurosciences, University of Cambridge, Cambridge CB20QQ, UK; mt628@medschl.cam.ac.uk (M.T.); hsm32@medschl.cam.ac.uk (H.S.M.)

**Keywords:** Mendelian randomization, single nucleotide polymorphisms, stroke, vitamin K1

## Abstract

Vitamin K plays a crucial role in blood coagulation, and hypercoagulability has been linked to atherosclerosis-related vascular disease. We used the Mendelian randomization study design to examine whether circulating vitamin K_1_ (phylloquinone) levels are associated with ischemic stroke. Four single-nucleotide polymorphisms associated with vitamin K_1_ levels were used as instrumental variables. Summary-level data for large artery atherosclerotic stroke (*n* = 4373 cases), small vessel stroke (*n* = 5386 cases), cardioembolic stroke (*n* = 7193 cases), and any ischemic stroke (*n* = 34,217 cases and 404,630 non-cases) were available from the MEGASTROKE consortium. Genetically-predicted circulating vitamin K_1_ levels were associated with large artery atherosclerotic stroke but not with any other subtypes or ischemic stroke as a whole. The odds ratios per genetically predicted one nmol/L increase in natural log-transformed vitamin K_1_ levels were 1.31 (95% confidence interval (CI) 1.12–1.53; *p* = 7.0 × 10^−4^) for large artery atherosclerotic stroke, 0.98 (95% CI 0.85–1.12; *p* = 0.73) for small vessel stroke, 1.01 (95% CI 0.90–1.14; *p* = 0.84) for cardioembolic stroke, and 1.05 (95% CI 0.99–1.11; *p* = 0.11) for any ischemic stroke. These findings indicate that genetic predisposition to higher circulating vitamin K_1_ levels is associated with an increased risk of large artery atherosclerotic stroke.

## 1. Introduction

Vitamin K is a group of fat-soluble vitamins that occur in two natural active forms. Vitamin K_1_ (phylloquinone) is present predominantly in green leafy vegetables and plant oils and is the main form of vitamin K in the Western diet [1,2]. Vitamin K_2_ (menaquinone) occurs in animal products (e.g., egg yolks and butter) and fermented foods (e.g., cheese), and is synthesized by certain intestinal bacteria [2]. Vitamin K_1_ is preferentially accumulated in the liver, where it plays an essential role in the synthesis of coagulation factors [3]. Warfarin, a commonly prescribed drug to control blood coagulation and to reduce thrombotic events, is a vitamin K antagonist. Contrary to vitamin K_1_, vitamin K_2_ has a more widespread distribution pattern [3] and is hypothesized to play a role in preventing cardiovascular disease by inhibiting vascular calcification [2]. Whereas a high vitamin K_1_ intake has been shown to increase arterial thrombosis tendency, vitamin K_2_ has been found to have the opposite effect [4].

Hypercoagulability tends to increase atherosclerosis and is linked to atherosclerosis-related ischemic arterial disease [5]. High plasma vitamin K_1_ levels were found to be associated with an increased prevalence of coronary artery calcification [6]. Thus, elevated circulating vitamin K_1_ levels, due to a high dietary intake or genetic predisposition, might increase the risk of cardiovascular disease. Observational studies investigating the association between dietary vitamin K_1_ intake and risk of cardiovascular disease are scarce and available studies show either no association or an inverse association [7,8,9,10]. A limitation of the observational findings is the potential concern of residual confounding, since a high vitamin K_1_ intake may reflect a healthy diet and lifestyle. Moreover, no study examined whether the association of vitamin K_1_ intake with ischemic stroke differs by ischemic stroke subtypes. 

**Figure 1:**
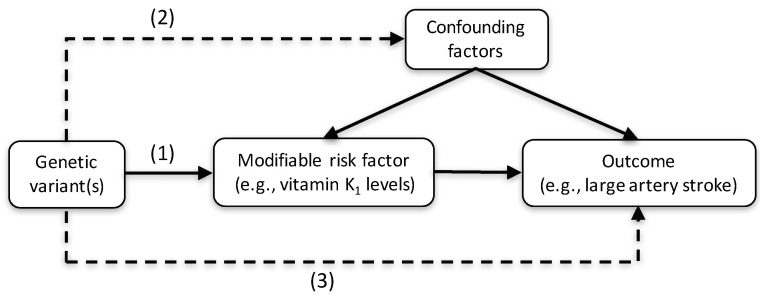
Concept of a Mendelian randomization (MR) study. A genetic variant (or multiple genetic variants in combination) associated with a modifiable risk factor, such as circulating vitamin K levels, can be used as an instrumental variable to determine the association between the risk factor and the outcome. The genetic variant used as an instrumental variable must (1) be robustly associated with the risk factor under study, (2) not be associated with any confounding factors of the risk factor-outcome relationship, and (3) affect the outcome through the risk factor under study and not through any other causal pathway. The dashed lines represent potential associations between variables that would represent violations of the Mendelian randomization assumptions.

Mendelian randomization (MR) is a technique that uses genetic variants associated with a modifiable (non-genetic) exposure of interest as proxy indicators (instrumental variables) for the exposure to infer causality [11]. Genetic variants are randomly assorted at meiosis and are thus less likely to be associated with potential confounding factors of the exposure-outcome relationship (Figure 1). A recent MR study showed that genetically higher vitamin K_1_ levels were associated with a significantly increased risk of coronary artery disease (odds ratio of 1.17 per 1 nmol/L increase of natural log-transformed vitamin K_1_ levels) [12], which is usually caused by atherosclerosis. It is unknown whether high circulating vitamin K_1_ levels increase the risk of large artery atherosclerotic stroke (LAS), which share several modifiable risk factors (e.g., hypertension, high-density lipoprotein cholesterol levels, and type 2 diabetes) [13,14,15] and non-modifiable genetic predisposition [16] with coronary artery disease.

We conducted a two-sample MR study to test the hypothesis that high circulating vitamin K_1_ levels are associated with an increased risk of LAS. We also examined the associations of genetically-predicted vitamin K_1_ levels with small-vessel stroke (SVS), cardioembolic stroke (CES), and ischemic stroke as a whole.

## 2. Methods

### 2.1. Data Sources and SNP Selection

A genome-wide association study of 2138 individuals of European ancestry identified 11 single-nucleotide polymorphisms (SNPs) in five loci that were associated with circulating vitamin K_1_ levels at *p* < 1 × 10^−6^ [17]. From each locus, we selected the SNP with the strongest association (lowest *p* value) with vitamin K_1_ levels. One of the SNPs (rs964184 in *ZNF259*) is strongly associated with cholesterol and triglyceride levels [18] and was excluded from the analyses to avoid pleiotropic bias. Thus, four SNPs were used as instrumental variables for vitamin K_1_ levels (Table 1).

Summary-level data for the four vitamin K_1_-associated SNPs with ischemic stroke and its subtypes were available from 438,847 European-descent individuals, including 34,217 cases of ischemic stroke, included in the MEGASTROKE consortium [19]. Subtyping of ischemic stroke was largely based on the Trial of Org 10,172 in Acute Stroke Treatment criteria. Data on ischemic stroke subtypes were available for 4373 LAS, 5386 SVS, and 7193 CES cases [19]. 

### 2.2. Statistical Analysis

The statistical analyses were conducted using the mrrobust package for Stata (StataCorp, College Station, TX, USA) [20]. For each of the four SNPs, we computed an instrumental variable ratio estimate by dividing the beta-coefficients (log odds ratio) for the SNP—stroke association by the beta coefficient for the SNP—vitamin K_1_ association. An overall odds ratio across the four SNPs was obtained by combining the ratio estimates using the inverse-variance weighted method (hereafter referred to as standard MR analysis) [21]. As sensitivity analyses, we used the weighted median method, which provides a valid estimate if at least 50% of weight comes from valid instrumental variables, and MR-Egger methods, which can explore and adjust for pleiotropy [21]. All statistical tests were two-sided, and considered statistically significant at a pre-specified *p* value below 0.05.

## 3. Results

**Figure 2:**
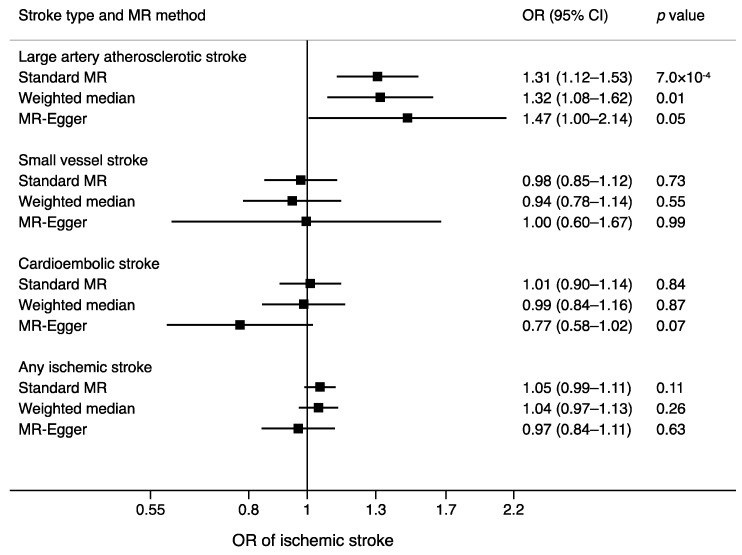
Associations of genetically predicted 1 nmol/L increase in natural log-transformed vitamin K_1_ levels with ischemic stroke subtypes and any ischemic stroke in different MR analyses. CI, confidence interval; MR, Mendelian randomization; OR, odds ratio.

Relationships of the vitamin K_1_-associated SNPs with ischemic stroke subtypes are shown in Table 1. Genetically-predicted circulating vitamin K_1_ levels were positively associated with LAS (Figure 2, Figures S1 and S2) but not with the other ischemic stroke subtypes or overall ischemic stroke (Figure 2). In the standard MR analysis, the odds ratios per genetically-predicted one nmol/L increase in natural log-transformed vitamin K_1_ levels were 1.31 (95% confidence interval (CI) 1.12–1.53; *p* = 7.0 × 10^−4^) for LAS, 0.98 (95% CI 0.85–1.12; *p* = 0.73) for SVS, 1.01 (95% CI 0.90–1.14; *p* = 0.84) for CES, and 1.05 (95% CI 0.99–1.11; *p* = 0.11) for any ischemic stroke (Figure 2). There was no heterogeneity between individual SNPs associations with LAS (*I*^2^ = 0%; *p* = 0.57; Figure S1).

Results were similar in the sensitivity analyses using the weighted median and MR-Egger methods but the CIs were broader, indicating lower power in these analyses compared to the inverse-variance weighted analysis (Figure 2). The MR-Egger analysis revealed no evidence of directional pleiotropy in the analysis of LAS (*p* = 0.52), SVS (*p* = 0.93), or any ischemic stroke (*p* = 0.20), but there was suggestive evidence of directional pleiotropy in the analysis of CES (*p* = 0.04).

We next searched the PhenoScanner database [22] for possible pleiotropic associations of individual SNPs with risk factors for LAS. Associations of the vitamin K_1_-associated SNPs were observed for rs4645543 with former smoking (*p* = 0.004), rs2108622 with diastolic blood pressure (*p* = 0.015) and circulating vitamin E levels (*p* = 1.4 × 10^−10^; the vitamin K_1_-increasing allele is associated with higher vitamin E levels), and rs2192574 with low-density lipoprotein cholesterol (*p* = 0.02). In a sensitivity analysis removing these SNPs, the OR of LAS ranged from 1.22 (95% CI 1.00–1.48; *p* = 0.047) when excluding rs2192574, to 1.36 (95% CI 1.15–1.62; *p* = 4.4 × 10^−4^) when excluding rs2108622.

## 4. Discussion

This study showed that genetically higher circulating vitamin K_1_ levels are associated with an increased risk of LAS. This finding, along with results from previous MR and cohort studies showing positive associations of circulating vitamin K_1_ levels with coronary artery disease [12] and calcification [6], suggests that high vitamin K_1_ levels may increase the risk of atherosclerotic-related vascular disease.

We observed no association of genetically-predicted vitamin K_1_ levels with other stroke subtypes or with overall ischemic stroke. The lack of association with overall ischemic stroke is consistent with results of previous observational studies showing no association between dietary vitamin K_1_ intake and overall risk of ischemic stroke [7,9,10]. Results of vitamin K_1_ intake in relation to LAS and other subtypes were not available in other observational studies.

Previous studies reported that genetic variants related to increased thrombosis increases risk of both LVS and CES, i.e., all subtypes with a presumed thromboembolic basis [23]. Therefore, in some aspects, an association between genetically higher vitamin K_1_ levels and CES might have been expected. We cannot exclude that we may have overlooked a weak association between genetically higher vitamin K_1_ levels and CES. However, an alternative explanation is that vitamin K_1_ may increase LAS risk by other mechanisms, including atherogenesis itself, and vitamin K_1_ has been related to increased arterial calcification [6]. Vitamin K is required for the conversion of coagulation factors II, VII, IX, and X into their biologically active forms. In a study of 600 ischemic stroke cases and 600 population-based controls, elevated factor VII antigen plasma levels were positively associated with risk of LAS but not CES [24].

An important strength of the present study is that confounding and reverse causality was mitigated through the use of genetic variants as proxies for circulating vitamin K_1_ levels. Two of the main potential biases that can affect the results of an MR study are population stratification (i.e., confounding by ancestry) and pleiotropy. Our findings are unlikely confounded by population stratification because the analyses included individuals of European ancestry only. Furthermore, our results were consistent in sensitivity analyses and there was no evidence that pleiotropy affected the results. However, when assessing pleiotropic associations of individual SNPs, we found that the SNP in the *CYP4F2* gene was associated with higher vitamin E levels. Although the role of vitamin E in LAS is unknown, our findings became somewhat stronger after exclusion of that SNP.

A limitation of this MR study is that the vitamin K_1_-asociated SNPs did not achieve conventional genome-wide significance (*p* < 5 × 10^−8^), probably due to the relatively small genome-wide association study [17]. This raises the possibility of false positives (i.e., type I error), and it cannot be ruled out that the SNPs associated with vitamin K_1_ levels are proxies for other causal SNPs. The standard MR approach assumes a linear association between the exposure and outcome. We could not examine if the association between vitamin K_1_ levels and LAS is non-linear and if there is a threshold effect. Another shortcoming is that we were unable to assess the association between circulating vitamin K_2_ (menaquinone) levels and ischemic stroke, as no SNPs has, to the best of our knowledge, been identified to be associated with vitamin K_2_ levels.

## 5. Conclusions

The findings of this MR study indicate that genetic predisposition to higher circulating vitamin K_1_ levels may be associated with an increased risk of LAS but no other ischemic stroke subtypes or with overall ischemic stroke. This finding warrant replication by further studies.

## Figures and Tables

**Table 1:** Characteristics of the vitamin K_1_-associated single-nucleotide polymorphisms and their associations with ischemic stroke subtypes.

				Vitamin K_1_		LAS		SVS		CES	
SNP	Nearby Gene	Chr	EA *	β ^†^	SE	β ^‡^	SE	β ^‡^	SE	β ^‡^	SE
rs4645543	*KCNK9*	8	C	0.42	0.08	0.089	0.060	−0.081	0.052	−0.050	0.044
rs2108622	*CYP4F2*	19	T	0.16	0.03	0.015	0.029	−0.023	0.026	0.042	0.021
rs2192574	*CTNAA2*	2	C	0.28	0.06	0.110	0.037	0.050	0.035	0.002	0.029
rs4122275§	*CDO1*§	5	G	0.68	0.17	0.220	0.156	−0.070	0.121	0.011	0.123

Abbreviations: CES, cardioembolic stroke; Chr, chromosome; EA, effect allele; LAS, large artery atherosclerotic stroke; SE, standard error; SNP, single nucleotide polymorphism; SVS, small vessel stroke. * Allele associated with higher vitamin K_1_ levels. ^†^ The β coefficient represents the change in vitamin K1 (ln-nmol/L) per additional copy of the effect allele, and is adjusted for age, sex, and study-specific covariates (e.g., study site, population stratification by principal components). ^‡^ Log odds ratio of stroke per additional copy of the effect allele. § The two SNPs in this locus with the strongest association with vitamin K_1_ levels were not available in the stroke dataset. The third strongest SNP (rs4122275) at this locus was available and used.

## Supplementary Materials

The following are available online at http://www.mdpi.com/2072-6643/10/11/1575/s1: Figure S1: Association of genetically predicted circulating vitamin K_1_ levels with large artery atherosclerotic stroke; Figure S2: Scatter plot of the four vitamin K_1_-associated single-nucleotide polymorphisms (SNPs) with circulating vitamin K_1_ levels and large artery atherosclerotic stroke (LAS); MEGASTROKE consortium authors.

## References

[1] Booth S.L., Suttie J.W. (1998). Dietary intake and adequacy of vitamin K. J. Nutr..

[2] Schwalfenberg G.K. (2017). Vitamins K1 and K2: The emerging group of vitamins required for human health. J. Nutr. Metab..

[3] Schurgers L.J., Vermeer C. (2002). Differential lipoprotein transport pathways of K-vitamins in healthy subjects. Biochim. Biophys. Acta Gen. Subj..

[4] Ronden J.E., Groenen-van Dooren M.M., Hornstra G., Vermeer C. (1997). Modulation of arterial thrombosis tendency in rats by vitamin K and its side chains. Atherosclerosis.

[5] Loeffen R., Spronk H.M., ten Cate H. (2012). The impact of blood coagulability on atherosclerosis and cardiovascular disease. JTH.

[6] Dalmeijer G.W., van der Schouw Y.T., Booth S.L., de Jong P.A., Beulens J.W. (2014). Phylloquinone concentrations and the risk of vascular calcification in healthy women. Arter. Thromb. Vasc. Biol..

[7] Erkkila A.T., Booth S.L., Hu F.B., Jacques P.F., Manson J.E., Rexrode K.M., Stampfer M.J., Lichtenstein A.H. (2005). Phylloquinone intake as a marker for coronary heart disease risk but not stroke in women. Eur. J. Clin. Nutr..

[8] Juanola-Falgarona M., Salas-Salvado J., Martinez-Gonzalez M.A., Corella D., Estruch R., Ros E., Fito M., Aros F., Gomez-Gracia E., Fiol M. (2014). Dietary intake of vitamin K is inversely associated with mortality risk. J. Nutr..

[9] Erkkila A.T., Booth S.L., Hu F.B., Jacques P.F., Lichtenstein A.H. (2007). Phylloquinone intake and risk of cardiovascular diseases in men. Nutr. Metab. Cardiovasc. Dis..

[10] Vissers L.E., Dalmeijer G.W., Boer J.M., Monique Verschuren W.M., van der Schouw Y.T., Beulens J.W. (2013). Intake of dietary phylloquinone and menaquinones and risk of stroke. J. Am. Heart Assoc..

[11] Smith G.D., Ebrahim S. (2003). ‘Mendelian randomization’: Can genetic epidemiology contribute to understanding environmental determinants of disease?. Int. J. Epidemiol..

[12] Schooling C.M. (2016). Plasma levels of vitamin K and the risk of ischemic heart disease: A Mendelian randomization study. JTH.

[13] Yusuf S., Hawken S., Ounpuu S., Dans T., Avezum A., Lanas F., McQueen M., Budaj A., Pais P., Varigos J. (2004). Effect of potentially modifiable risk factors associated with myocardial infarction in 52 countries (the INTERHEART study): Case-control study. Lancet.

[14] Larsson S.C., Scott R.A., Traylor M., Langenberg C.C., Hindy G., Melander O., Orho-Melander M., Seshadri S., Wareham N.J., Markus H.S. (2017). Type 2 diabetes, glucose, insulin, BMI, and ischemic stroke subtypes: Mendelian randomization study. Neurology.

[15] Hindy G., Engstrom G., Larsson S.C., Traylor M., Markus H.S., Melander O., Orho-Melander M. (2018). Role of blood lipids in the development of ischemic stroke and its subtypes: A Mendelian randomization study. Stroke.

[16] Kessler T., Erdmann J., Dichgans M., Schunkert H. (2015). Shared genetic aetiology of coronary artery disease and atherosclerotic stroke—2015. Curr. Atheroscler. Rep..

[17] Dashti H.S., Shea M.K., Smith C.E., Tanaka T., Hruby A., Richardson K., Wang T.J., Nalls M.A., Guo X., Liu Y. (2014). Meta-analysis of genome-wide association studies for circulating phylloquinone concentrations. Am. J. Clin. Nutr..

[18] Willer C.J., Schmidt E.M., Sengupta S., Peloso G.M., Gustafsson S., Kanoni S., Ganna A., Chen J., Buchkovich M.L., Mora S. (2013). Discovery and refinement of loci associated with lipid levels. Nat. Genet..

[19] Malik R., Chauhan G., Traylor M., Sargurupremraj M., Okada Y., Mishra A., Rutten-Jacobs L., Giese A.K., van der Laan S.W., Gretarsdottir S. Multiancestry genome-wide association study of 520,000 subjects identifies 32 loci associated with stroke and stroke subtypes. Nat. Genet..

[20] Spiller W., Davies N.M., Palmer T.M. (2017). Software application profile: Mrrobust—A tool for performing two-sample summary Mendelian randomization analyses. BioRxiv.

[21] Burgess S., Bowden J., Fall T., Ingelsson E., Thompson S.G. (2017). Sensitivity analyses for robust causal inference from Mendelian randomization analyses with multiple genetic variants. Epidemiology.

[22] Staley J.R., Blackshaw J., Kamat M.A., Ellis S., Surendran P., Sun B.B., Paul D.S., Freitag D., Burgess S., Danesh J. (2016). PhenoScanner: A database of human genotype-phenotype associations. Bioinformatics.

[23] Williams F.M., Carter A.M., Hysi P.G., Surdulescu G., Hodgkiss D., Soranzo N., Traylor M., Bevan S., Dichgans M., Rothwell P.M. (2013). Ischemic stroke is associated with the ABO locus: The EuroCLOT study. Ann. Neurol..

[24] Stanne T.M., Hanson E., Olsson S., Hoglund J., Jood K., Blomstrand C., Jern C. (2013). Factor VII antigen levels are differentially associated to etiological subtypes of ischaemic stroke. Thromb. Haemost..

